# The Response of Macro- and Micronutrient Nutrient Status and Biochemical Processes in Rats Fed on a Diet with Selenium-Enriched Defatted Rapeseed and/or Vitamin E Supplementation

**DOI:** 10.1155/2017/6759810

**Published:** 2017-05-30

**Authors:** Michaela Rýdlová, Karolína Růnová, Jiřina Száková, Alena Fučíková, Anna Hakenová, Petr Mlejnek, Václav Zídek, Jana Tremlová, Oto Mestek, Antonín Kaňa, Jarmila Zídková, Magdalena Melčová, Klára Truhlářová, Pavel Tlustoš

**Affiliations:** ^1^Faculty of Agrobiology, Food and Natural Resources, Czech University of Life Sciences in Prague, Prague, Czech Republic; ^2^Institute of Physiology, Academy of Science of the Czech Republic, Prague, Czech Republic; ^3^Faculty of Chemical Engineering, University of Chemistry and Technology, Prague, Czech Republic; ^4^Faculty of Food and Biochemical Technology, University of Chemistry and Technology, Prague, Czech Republic

## Abstract

The response of nutrient status and biochemical processes in (i) Wistar and (ii) spontaneously hypertensive (SHR) rats upon dietary intake of selenium- (Se-) enriched defatted rapeseed (DRS) and/or vitamin E fortification was examined to assess the health benefit of DRS in animal nutrition. Twenty-four individuals of each type of rat were used: The control group was fed with an untreated diet (Diet A). In Diets B and C, soybean meal was replaced with defatted DRS, which comprised 14% of the total diet. The selenized DRS application resulted in ~3-fold increase of Se content in the diet. Diet C was also fortified with the addition of vitamin E, increasing the natural content by 30%. The Se content of the blood and kidneys tended to increase in the DRS groups, where the changes were significant (*P* < 0.05) only in the case of SHR rats. The iodine (I) content and the proportion of iodide in rat livers indicated a lower transformation rate of iodide into organoiodine compounds compared to the control. Slight and ambiguous alterations in the antioxidative response of the rat were observed in the DRS groups, but the addition of vitamin E to the diet helped to moderate these effects.

## 1. Introduction

Selenium (Se) is a component of more than 30 selenoproteins, which play a significant role in humans and other animals. Selenoproteins protect cells from damage inflicted by free radicals, participate in the metabolism of thyroid hormones, control reproductive functions, and stimulate the immune system [1, 2]. Lukas et al. [3] reported a high and increasing incidence of thyroid cancer in the Czech Republic. Among the factors affecting this incidence is insufficient dietary intake of iodine (I) and, to a lesser extent, selenium. Selenium and iodine are essential for thyroid hormone synthesis and function. Thus, synthesis of thyroid hormones and their activation and inactivation by deiodinase enzymes require Se. Wu et al. [4] confirmed that low selenium status is associated with an increased risk of thyroid disease, and, therefore, increased selenium intake may reduce the risk in areas where selenium uptake is low. In Europe, Wimmer et al. [5] reported slightly higher (but not significant) serum selenium levels in normal people than in patients with autoimmune thyroiditis in Lower Austria. A negative correlation between the thyroid volume and Se and I content of hair of goitrous children in Morocco was observed by El-Fadeli et al. [6].

As reviewed by Loyke [7] and Mozaffarian [1], selenium is involved in defense against cardiovascular diseases in animals. Selenium belongs to a group of trace elements that includes iron, copper, zinc, and manganese. They are essential components of enzymatic systems that can affect arterial hypertension and myocardial infarction [8, 9]. Other nutritional factors that affect blood pressure and cardio- and cerebrovascular diseases include sodium, potassium, calcium, magnesium, vitamins A, C, and E, and essential fatty acids [10]. Antioxidant defenses that are affected by selenium may reduce vascular and tissue injury resulting from the formation of reactive oxygen species due to stress, hypoxia, hypertension, hyperlipidemia, and diabetes. Selenium-related systems may also decrease the oxidation of lipids and protect the vascular endothelium from damage. Conversely, extremely high selenium status can lead to increased blood pressure [11]. In a review of previously published work, it was found that the relationship between Se status and hypertension is still ambiguous [12].

The role of selenium in animals is supported by vitamin E [2]. Thakur and Srivastava [13] extensively reviewed the roles of vitamin E in humans, which included acting as an antioxidant, supporting the metabolism of nucleic acids, protein, and lipids, and helping to protect against heart disease. Moreover, vitamin E is involved in the protection of thyroid functions. Yu et al. [14] showed that excess iodine in the diet leads to thyroid damage in rats and vitamin E supplementation can partly ameliorate iodine-induced thyroid cytotoxicity.

Selenium concentration in cattle whole blood samples collected from 494 animals within the Czech Republic ranged between 0.004 and 0.212 mg/L [15]. Selenium deficiency was found in 50% of the tested animals and on 54% of the farms [15]. Fortification of nutritional resources can lead to an improved Se status in humans and other animals. Bañuelos and Mayland [16] showed that selenized rapeseed* (Brassica napus)* plants (not including seeds) could be safely fed to lambs and cows to help meet normal Se intake requirements. However, rapeseed is characterized by a high content of glucosinolates. Glucosinolate intake has been found to decrease the utilization of iodine in the thyroid gland, resulting in low levels of triiodothyronine and thyroxine [17, 18]. In our previous experiments, Tvrdá et al. [19] found that the substitution of soybean* (Glycine max)* meal with 00-quality DRS (representing 14% of the total diet) in rat diets did not result in any harmful effects on the biochemical or hematological response of the rats. Thus, the potential antinutritional characteristics of DRS did not affect the main parameters of the biochemical response in rats. Myška et al. [20] tested the effect of an increased selenized DRS rate (up to 14% of the total diet) on the uptake of Se and other essential elements by male Wistar rats. They showed that the Se content in the blood and liver of the animals remained unchanged; increased Se levels were observed only in the kidneys of the selenized DRS group compared to the controls [20]. However, Se addition seems to help in the utilization of essential elements such as phosphorus, sulfur, and zinc, compared to the DRS-containing diet without Se fortification [20].

All the mentioned findings document interrelationships of the selenium, iodine, and vitamin E in both animal and human organism. Therefore, the Se supplementation of the animal diet can lead to both improvement of the Se status of the organism and/or of the physiological functions connected with iodine and vitamin E roles in the organism. Moreover, the potential increase of the Se content in farm animals (for instance pigs) can also improve the human Se status with all the other connections. In this study, the potential role of vitamin E addition in the uptake and utilization of Se, I, and other macro- and micronutrients by rats was investigated. The main objectives of the study were (i) to assess the potential mutual effect of Se and vitamin E on the utilization of macro- and micronutrients within DRS groups, (ii) to assess the potential effect of the selenized DRS on iodine utilization, and (iii) to verify the potential effect of Se and/or vitamin E dietary intake on hypertensive rats compared to normotensive rats.

## 2. Materials and Methods

### 2.1. Experimental Design

Male Wistar Kyoto rats and spontaneously hypertensive rats (SHR) were obtained from a breeder (Velaz, Prague, Czech Republic) at 30 days of age and housed in cages (one animal per cage) in a room with a controlled temperature (varying from 23 to 25°C) under natural light conditions. Twenty-four animals of each strain, divided into three groups of eight, were used; the animals were fed on the semisynthetic diet according to the experimental design for 60 days. Feed and water were supplied to the animals ad libitum. Feed consumption and body weight of the animals were monitored weekly. The control group was fed with the untreated semisynthetic diet (Diet A). In the case of DRS-treated groups (Diets B and C), the complete portion (14% of the whole diet) of soybean meal was replaced with Se-enriched DRS. The DRS was prepared from oilseed rape of the 00-quality variety, NK Oktans, where the elemental composition of the seeds was as follows: 9159 ± 1837 mg/kg of Ca, 2.63 ± 0.3 mg/kg of Cu, 73.0 ± 13.1 mg/kg of Fe, 11054 ± 2688 mg/kg of K, 1273 ± 195 mg/kg of Mg, 20.4 ± 4.5 mg/kg of Mn, 3067 ± 341 mg/kg of P, 2533 ± 765 mg/kg of S, and 15.7 ± 2.1 mg/kg of Zn [21]. The Se enrichment of the oilseed rape plants was provided by the foliar application of selenate (Na_2_SeO_4_) solution at the rate 50*g* Se/ha at the beginning of stem elongation in a microscale field experiment. The seeds were processed by milling and defatting the seeds using a Soxhlet apparatus and using hexane as the extraction agent for four hours. Subsequently, the meal was dried at 105°C for two hours and homogenized. The nutritional characteristics of the final DRS were as follows: 30.5% protein, 2.5% lipid, 18.4% metabolizable saccharides, 35.4% dietary fiber, 6.7% ash, 6.5% water, and 938 kJ/100*g* metabolizable energy [20]. Additionally, Diet C was fortified with vitamin E where the useable content was increased by 30% to enhance the vitamin E uptake within the physiological requirements of the animals. The composition and nutritional values are summarized in Table 1. At the end of the study period, the animals were euthanised by exsanguination after being anesthetized with Xylapan (xylazine) and Narketan (ketamine), and whole blood, liver, kidney, and testes were sampled. The sampled tissues were kept at −18°C and subsequently freeze-dried and homogenized; aliquots of blood samples were stored in heparinized tubes.

### 2.2. Total Element Content in Tissue Samples and Experimental Diets

In order to determine the quantity of elements in the freeze-dried and homogenized animal tissues and diets, an aliquot (~500 mg of dry matter) of the sample was weighed in a digestion vessel. In the case of blood, 500 *μ*L of whole blood was measured out into the digestion vessel. Concentrated nitric acid (8.0 mL, Analytika Ltd., Czech Republic) and 30% H_2_O_2_ (2.0 mL, Analytika Ltd., Czech Republic) were added. The mixture was heated in an Ethos 1 (MLS GmbH, Germany) microwave-assisted wet digestion system for 30 min at 220°C. After cooling, the digest was transferred to a 25 ml glass tube, topped up with deionized water, and kept at laboratory temperature until measurements were taken. For the element measurements of whole blood, the same decomposition procedure was applied, where 0.3 mL of the whole blood sample was taken for the analysis.

To determine total iodine quantities in the experimental diets, alkaline digestion of the liver and thyroid gland tissues was performed with 25% (w/w) solution of tetramethylammonium hydroxide pentahydrate (TMAH) prepared by dissolution of solid TMAH in water (Sigma-Aldrich, Steinheim, Germany). The samples (0.5–1*g*) were decomposed using microwave digestion with 3 mL of 25% (w/w) TMAH solution in 210 mL PTFE vessels in the decomposition unit, UniClever (Plazmatronika, Wroclaw, Poland) (digestion program: max. power 90 W for 1 min, max. power 105 W for 1 min, max. power 120 W for 1 min, max. power 135 W for 7 min, and 10 min cooling). After cooling, the samples were transferred into 25 mL volumetric flasks. These solutions were filtered through 0.45 *μ*m filters (Whatman, Buckinghamshire, UK); 9 mL of the filtrates was pipetted into 10 mL volumetric flasks, 0.2 mL of the internal standard (IS) solution was added, and the flasks were refilled with water to the mark before the measurements. The TMAH extraction is known to be a suitable method for sample preparation for iodine determination in both animal and plant materials [22].

Se content of the digests was measured by inductively coupled plasma mass spectrometry (ICP-MS, Agilent 7700x, Agilent Technologies Inc., USA). The ICP-MS was equipped with an autosampler ASX-500, a three-channel peristaltic pump, and a MicroMist nebulizer. The experimental conditions were as follows: RF power of 1.55 kW; plasma flow of 15.0 L/min; auxiliary flow of 0.9 L/min; helium collision cell flow of 8 L/min. Inductively coupled plasma-atomic emission spectrometry (ICP-OES, Agilent 720, Agilent Technologies Inc., USA), equipped with a two-channel peristaltic pump, a Sturman–Masters spray chamber, and a V-groove pneumatic nebulizer made of inert material, was applied for the determination of cadmium, copper, iron, manganese, zinc, phosphorus, and sulfur levels in the digests. The experimental conditions were as follows: power of 1.2 kW; plasma flow of 15.0 L/min; auxiliary flow of 0.75 L/min; nebulizer flow of 0.9 L/min. Flame atomic absorption spectrometry (F-AAS, Varian 280FS, Varian, Australia; air flow of 13.5 L/min, acetylene flow of 2.2 L/min, burner height of 13.5 mm, and nebulizer uptake rate of 5 mL/min) was used for the determination of Ca, Mg, and K quantities in the digests.

To establish total iodine concentrations in the alkaline digests, ICP-MS was applied using an ELAN DRC-e instrument (Perkin Elmer, Concord, Canada). The measurement conditions were as follows: RF power 1.1 kW; nebulizer gas flow rate 0.76 L/min; auxiliary gas flow rate 1 L/min; plasma gas flow rate 11 L/min; measured isotopes were ^72^Ge and ^127^I. The procedures were performed according to Kaňa et al. [23]. A solution of approximately 1% (w/w) TMAH served as a washing solution between measurements of the samples to prevent memory effects. Because of the excellent linearity of ICP-MS, a calibration curve based on a blank solution and a single calibration solution was sufficient. The limit of detection was estimated by tripling the standard deviation of the blanks (*n* = 6); its value was 0.97 *μ*g/L I (26.9 *μ*g/kg I for 1*g* of sample).

### 2.3. Iodine Speciation in Rat Liver

For the speciation analysis, the thawed samples (approx. 1*g*) were homogenized using Ultra-Turrax® T10 basic disperser with an S10N-10G dispersing element (IKA, Germany), with 2 mL of demineralized water for 1 min at 20,500 rpm. Solid particles were separated using centrifugation for 20 min at 10,000 rpm, and the supernatant was transferred to a 10 mL volumetric flask. The solid particles were shaken with 2 mL of water and the suspension was centrifuged. The combined extract was refilled to the mark with water and the solutions were filtered through 0.45 *μ*m filters before analysis. This filtered extract was also used to determine the total iodine content.

A high-pressure pump, Series 200 (Perkin Elmer, Shelton, USA), was used for the mobile phase delivery to the chromatographic column. The chromatographic column PRP ×100 (250 × 4.6 mm, 5 *μ*m, Hamilton) was used for species separation. The samples and standards were injected via Rheodyne 9010 injectors equipped with 50 *μ*L PEEK sample loops. The effluent from the column was mixed with an internal standard solution using a T-piece. The mixture was delivered into ICP-MS equipment using a peristaltic pump. The solution uptake was 1.0 mL/min of column effluent + 0.3 mL/min of internal standard solution. ICP-MS measurements were carried out using an ELAN DRC-e instrument (Perkin Elmer, Concord, Canada). A standard solution of 1000 ± 2 mg L^−1^ germanium (Ge, CertiPur, Merck, Darmstadt, Germany) was used for the preparation of a stock solution of the internal standard containing 5 mg L^−1^ Ge (IS). A standard solution of iodide (1000 ± 2 mg L^−1^ I, Analytika, Prague, Czech Republic) and solid potassium iodate (GR ACS, Merck, Darmstadt, Germany) were used for preparation of the calibration solutions. Demineralized water, Milli-Q (MilliPore, Bedford, MA, USA), was used for the preparation of all solutions. Solution of 100 mmol L^−1^ ammonium nitrate (extra pure, Merck, Darmstadt, Germany) served as the mobile phase for chromatography; the pH was adjusted via the addition of a 25% ammonia solution (SupraPur, Merck, Darmstadt, Germany) to a value of 7.4. The limits of detection and accuracy of determination were calculated using chromatography-ICP-MS coupling through the analysis of iodide and iodate solutions (*n* = 6) with low concentrations (both approximately 0.1 *μ*g/L I) and were estimated to be triple the standard deviation. The detection limit was 0.11 *μ*g/L (1.1 *μ*g/kg I for 1*g* of sample) for both iodide and iodate [23].

### 2.4. Determination of Hematological and Biochemical Parameters

The hematological parameters, total number of erythrocytes (Er, T.L-1), hemoglobin (Hb, g.100 mL-1), hematocrit value (Hct, %), mean cell volume (MCV, fL), and total number of leukocytes (Le, G.L-1), were determined in the whole blood that had been stabilized using K_2_EDTA. All parameters were determined using the computerized analyzer, NIHON KOHDEN MEK 5208. The blood hemolysis solution ISOTONAC 3 MEK 640 was used for the determination of the number of leukocytes and hemoglobin values. In the case of biochemical parameters, alanine aminotransferase (ALT) and cholesterol content in blood plasma were determined using the computerized analyzer, Cobas 6000 (Roche, Switzerland).

The activities of selected antioxidant enzymes such as glutathione peroxidase (GPx), glutathione reductase (GR), glutathione S-transferase (GST), thioredoxin reductase (TrxR), and catalase (CAT) were measured in extracts of rat liver and kidneys. The tissue extracts were prepared by the homogenization of 0.08*g* of liver or kidney with 1 mL of the buffer (0.01 mol·L^−1^ phosphate buffered saline solution at pH 7.4, 0.137 mol·L^−1^ NaCl, 8.1 mmol·L^−1^ Na_2_HPO_4_·12H_2_O, 1.5 mmol·L^−1^ KH_2_PO_4_, and 2.7 mmol·L^−1^ KCl). The homogenates were than centrifuged (Jouan, France) for 10 min at 6,700 ×g and 4°C and the supernatants were kept at −20°C until measurement. Specific enzymatic activity in the tissue extracts was determined using the spectrophotometers, Libra S22 (Biochrom, UK) and PowerWave XS (BioTek, USA). Catalase activity was determined according to Góth [24] by using the Catalase Assay Kit (Cayman Chemical, USA). The extracts were mixed with 50 mmol·L^−1^ Sörensen buffer (consisting of 19.1 mmol·L^−1^ Na_2_HPO_4_·12H_2_O and 30.9 mmol·L^−1^ KH_2_PO_4_) at pH 7.0 and measured at *λ* = 240 nm. Glutathione peroxidase activity was determined by the modified method published by Flohé and Günzler [25] in 100 mmol·L^−1^ potassium phosphate buffer with 1 mmol·L^−1^ EDTA at pH 7.0 and measured at *λ* = 340 nm. Glutathione reductase was determined according to Worthington and Rosemeyer [26] in 47 mmol·L^−1^ potassium phosphate buffer (pH = 7) at *λ* = 412 nm. Thioredoxin reductase was measured according to Luthman and Holmgren [27] 100 mmol·L^−1^ potassium phosphate buffer with 1 mmol·L^−1^ EDTA at pH 7.0, at *λ* = 412 nm. Glutathione S-transferase was measured according to Habig et al. [28] in the buffered saline solution (pH = 7.2) at *λ* = 340 nm. All the analyses were performed at 25°C.

### 2.5. Statistics

The data were processed using Microsoft Office Excel 2007 and Statistica 12 CZ statistical software. One-way analysis of variance (ANOVA) at *P* < 0.05 followed by Scheffé's test was applied to the data. The Shapiro-Wilk test (*P* < 0.05) was used to verify the normality of the data distribution. To assess the differences in the activities of the antioxidative enzymes among the experimental groups, the *F*-test was applied to observe whether the data complied with the assumption of homogeneity of variance. Subsequently, the unpaired two-sample *t*-test (*P* < 0.05), with a Holm–Bonferroni correction to counteract the problem of multiple comparisons, was applied.

## 3. Results

### 3.1. The Effect of Se and Vitamin E on the Utilization of Macro- and Micronutrients and Biochemical Parameters within DRS Groups

Table 1 documents no difference in the basic nutritional composition of the experimental diets. As presented in Table 2, the replacement of soybean meal with the selenized DRS resulted in an increase in total Se content (as expected), as well as an increase in the total potassium content in the diet, due to the high natural content of potassium in rapeseed. The other elements that were measured remained unchanged. Se concentrations in whole blood are summarized in Table 3. In most studies presenting blood Se levels, the plasma selenium values are used because plasma selenium responds rapidly to selenium supplementation and is regarded as a biomarker of short-term selenium status. However, whole blood selenium is used as a biomarker of long-term selenium intake and status [29], so whole blood Se concentration was the more suitable parameter in this experiment. No change in Se content was found in the liver (Table 4), but there was an increase in Se in the kidneys and testes (Tables 5 and 6). In these tissues, however, no significant differences were found among the Se-enhanced experimental diets. Thus, the enhancement of the vitamin E uptake (Diet C) did not result in the improvement of the Se utilization by the rats compared to Diet B. Among the other investigated elements, the blood levels of Ca, Cu, and P increased in the DRS groups of Wistar rats, and, on the contrary, except S, the blood levels of elements tended to decrease in the DRS groups of SHR rats. In the kidney, no alterations in element content were observed, except for ambiguous changes in S content (*P* < 0.05) in the Wistar rats. In the testes, no effect of the different diets was observed in the Wistar rats, whereas elevated (*P* < 0.05) contents of Mg, P, and S were determined in SHR rats.

**Figure 1:**
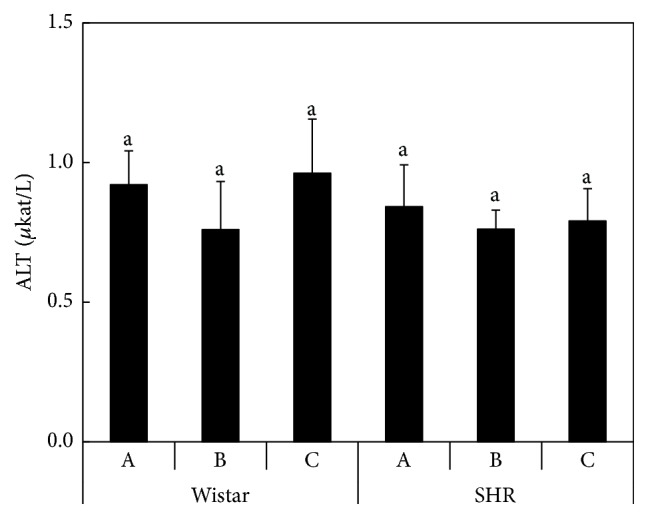
The ALT activity in blood plasma of rats (*μ*kat/L) according to the Diets A, B, and C; the averages marked by the same letter did not significantly differ at *P* < 0.05 within individual rat strains; data are presented as mean ± standard deviation, *n* = 8.

No significant differences (*P* < 0.05) in the main hematological parameters of rats were found due to the addition of selenized DRS to the diet (Table 7). In Wistar rat liver, GR, GST, and TrxR activity increased significantly (*P* < 0.05) in rats fed on the selenized DRS, and this effect was accentuated in Diet C that had elevated vitamin E level (Table 8). Thus, these results indicate potential interaction of Se and vitamin E in the antioxidative response of the rat organism. For GPx, the activity in the liver decreased (*P* < 0.05) with Se-DRS addition to the diet. CAT activity increased (*P* < 0.05) in the group fed on Diet B and increased (*P* < 0.05) again under Diet C. Another enzyme that may have indicated adverse effects of DRS on liver function was alanine aminotransferase (ALT), but no significant changes among the experimental groups were observed (Figure 1).

### 3.2. The Effect of Se and/or Vitamin E Dietary Intake on Se Utilization and Biochemical Parameters of Hypertensive Rats Compared to Normotensive Rats

The significantly higher (*P* < 0.05) kidney Se content was determined in the SHR rats fed on Diet C compared to Diet B (Table 5). The content of other investigated elements in the analyzed tissues showed differences in element uptake between Wistar and SHR rats (Tables 3–6), whereas the effect of the different diets on each individual rat strain was ambiguous. In whole blood, most of the elements showed lower values in SHR rats compared to Wistar rats, with the exception of manganese, which displayed the opposite pattern. SHR rats exhibited lower levels of most of the investigated elements in the liver, kidneys, and testes compared to Wistar rats.

**Figure 2:**
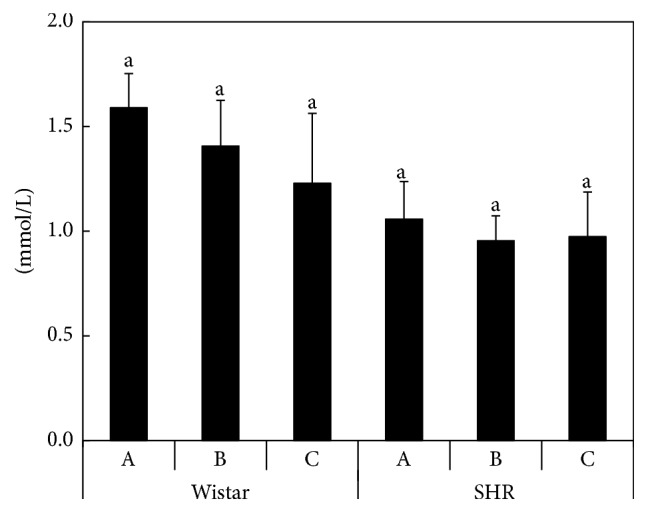
The cholesterol content in blood plasma of rats (mmol/L) according to the Diets A, B, and C; the averages marked by the same letter did not significantly differ at *P* < 0.05 within individual rat strains; data are presented as mean ± standard deviation, *n* = 8.

The average activity levels of the antioxidative enzymes in the liver and kidneys of SHR and Wistar rats are summarized in Table 8. In the liver of SHR rats, the activity of GPx, TrxR, and CAT decreased (*P* < 0.05) for Diet C, which was supplemented with Se and vitamin E. The levels of GR activity increased in the Se-supplemented Diet B and decreased again in the case of Diet C. In the kidneys of Wistar rats, a stepwise increase (*P* < 0.05) in GST activity in the order Diet A < Diet B < Diet C was found, whereas CAT activity remained unchanged. The activity of GPx and GR decreased in the group fed with Diet B and increased for Diet C with enhanced Se and vitamin E intake. TrxR activity increased in the group fed on Diet B and decreased for Diet C compared to the control (*P* < 0.05). In the kidneys of the SHR rats, the results were comparable to the Wistar rats, but without the decrease in GPx and GR activity seen in the Se-supplemented Diet B. Generally, significantly (*P* < 0.05) lower activity of GST was recorded in SHR rats compared to the Wistar strain in both the liver and kidneys, regardless of the experimental diet. Moreover, no differences in ALT activity were recorded between Wistar and SHR rats (Figure 1). The cholesterol content in blood plasma in this experiment (Figure 2) suggested a slight decreasing trend in plasma cholesterol content in Wistar rats (and to a lesser extent in SHR rats) fed on the DRS-containing Diets B and C, but the differences were not statistically significant.

### 3.3. Iodine Content and Speciation in Rats

**Figure 3:**
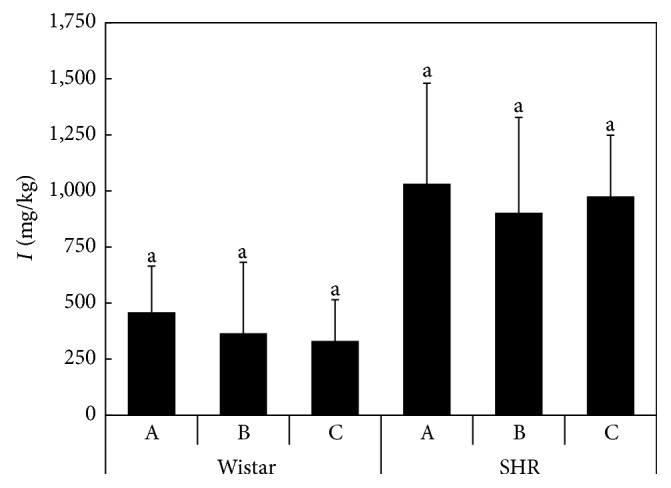
The total iodine contents in thyroid glands of rats (mg/kg) according to the Diets A, B, and C; the averages marked by the same letter did not significantly differ at *P* < 0.05 within individual rat strains; data are presented as mean ± standard deviation, *n* = 8.

**Figure 4:**
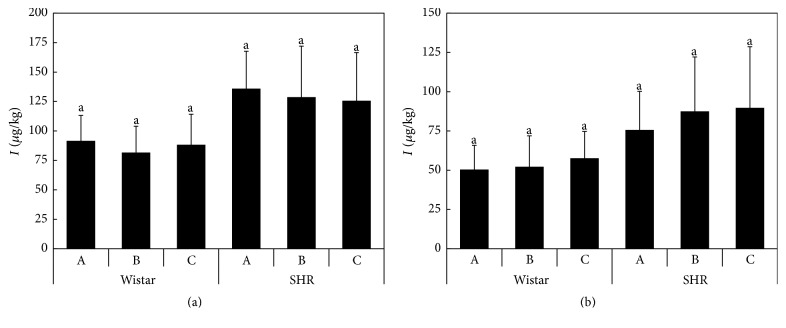
The total iodine (A) and iodide (B) contents in the extracts of the rat liver (*μ*g/kg) according to the Diets A, B, and C; the averages marked by the same letter did not significantly differ at *P* < 0.05 within individual rat strains; data are presented as mean ± standard deviation, *n* = 8.

More than 80% of total iodine content in mammals is concentrated in the thyroid [30]. The results of the total iodine content in whole thyroid glands are summarized in Figure 3. Regardless of the high variability of the results, SHR rats showed significantly higher iodine levels than the Wistar rats. However, no significant differences were observed within the individual strains. The average values were 200–275 mg/kg for Wistar rats and 541–620 mg/kg for SHR rats. The total amount of iodine and water-extractable iodine in the rat liver indicates no effect of elevated Se in the rat diet on iodine levels (Figure 4), as well as no effect of enhanced vitamin E uptake by the rats. Similarly, in the case of the thyroid glands, higher I content was determined in SHR rats compared to Wistar rats.

## 4. Discussion

Potassium is a major nutrient responsible for nitrogen economy in oilseed rape, formation of yield components, and, consequently, oilseed rape yield [31]. Brennan and Bolland [32] reported 32% higher potassium (K) requirements of oilseed rape than wheat* (Triticum aestivum)* for the production of maximum seed yield. At the same K application rate, the concentration of K in shoots was greater for rapeseed than for wheat [33]. However, soybean also has a high requirement of K in the soil compared to wheat [34]. Thus, we can assume that the differences in K content of the rat diet due to the replacement of soybean meal with DRS are caused predominantly by different growing conditions, soil K status, and mobility of both crops used in this experiment. For instance, it has been shown that soil pH affects K uptake by soybean plants [35]. Thus, among the investigated elements, the replacement of soybean meal with DRS in the rat diet can lead to increasing dietary uptake of K.

Higher Se levels in the diets resulted in increased blood Se levels, where the differences among the experimental diets were significant (*P* < 0.05) only in the case of SHR rats. In the blood of both animal strains, Se concentrations tended to increase if the vitamin E level was enhanced in the diet (Diet C). Generally, the SHR rats showed higher blood Se concentrations compared to Wistar rats, regardless of the diet. Similar findings were published by Loyke [36] in the serum of SHR rats compared to normotensive animals, suggesting different metabolism of the SHR rats. Yakobson et al. [8] compared NISAG (hereditary stress-induced arterial hypertension) rats and Wistar rats with acute myocardial infarction and found that, during recovery, selenium content in the plasma and myocardium of NISAG rats was higher than in normotensive animals, most probably due to more intensive functioning of the antioxidant systems. Negative correlations between both systolic and diastolic blood pressure and serum selenium levels were observed by Telišman et al. [37]. Similarly, Lymbury et al. [38] confirmed that high dietary Se intake (1000 mg Se per kg of the diet) resulted in lower levels of cardiac oxidative damage and a reduction in disease severity and mortality in SHR rats. In the present experiment, the Se content of the diet was lower than in the study of Lymbury et al. [38], but the data also indicate the stimulation of the antioxidative response.

Although the DRS addition into the rat diet led to higher uptake of K compared to control, no increase of K level in the animal tissues was determined in Wistar rats and even decrease of K was observed in the blood of SHR rats. Decreasing Zn and increasing Mn levels in serum of hypertensive patients were reported by Gouaref et al. [39], and positive relationships between blood Mn level and hypertension were observed by Vigeh et al. [40]. According to Martinez and Diaz [41], this positive interaction could be related to the role of manganese in erythropoietin synthesis. Alterations in tissue distribution of Fe, Mn, Cu, and Zn were documented by Giray et al. [42] in I- and/or Se-deficient rats. Under the elevated Se uptake conditions in this experiment, no changes in these elements were observed. In the rat liver, increased (*P* < 0.05) Zn contents were observed in the Wistar rats fed on Diet C with enhanced vitamin E. Lima et al. [43] reported high Zn content in the liver of lambs fed on a diet supplemented with vitamin E. Contrary to our results, they observed a decrease in Se and an increase in Cu levels in the serum [41], but these results are not comparable to our data as we measured whole blood, rather than serum. In our case, the blood Se content in SHR rats increased (*P* < 0.05) and Ca, Cu, Fe, K, Mg, P, and Zn decreased, whereas blood Ca and Cu concentrations in Wistar rats increased (*P* < 0.05) with increasing Se concentration. The hypertension is frequently associated with insulin resistance, and insulin can modify the functional activity of the hypothalamus [44], supporting the findings concerning the different metabolism of SHR rats compared to the normotensive animals [36]. Thus, the potential changes in nutrient utilization, including the essential elements, could be speculated in SHR rats as compared with the Wistar rats.

The total iodine content in whole thyroid glands (Figure 3) is substantially higher than in human thyroids, where total iodine in the whole glands is 3.44 ± 1.11 mg/kg [45]. In accordance with the previous results, only iodide was detected in the liver extracts (Figure 4), whereas the presence of iodate was not confirmed [23]. The iodide proportions in the extracts varied between 55 and 66% in Wistar and between 55 and 70% in SHR rat livers. Similar proportions of iodide were determined by Kaňa et al. [23] in porcine liver and turkey liver (48.2 ± 8.1% and 50.2 ± 6.5%, resp.). Although the differences were not significant, the results suggested a higher percentage of iodide in the liver of animals fed on the selenized diet, which indicates a lower transformation rate of iodide into organoiodine compounds, compared to the control.

High selenium content in the thyroid gland, even during selenium deficiency, has been reported by Schmutzler et al. [46]. The lowest selenium concentration for a single thyroid gland sample was 0.51 ± 0.05 mg/kg and the highest was 1.49 ± 0.20 mg/kg [45]. In our case, however, the Se content of the thyroid glands was not determined because of an insufficient amount of material for these analyses. Moreover, plasma or serum selenium concentrations do not reflect intrathyroid concentrations and cannot be used for the estimation of Se status in the thyroid [47]. Windisch [48] reviewed the available literature concerning the intake and interactions of essential trace elements and assumed that there is no homeostatic control of absorption of I. Rather, organisms seem to be passively exposed to influx of this element and the regulation of its metabolism is predominantly via renal excretion [48]. For these metabolic pathways, the element must be present in a metabolically recognizable form, and, for iodine, the major metabolic species is iodide.

Our previous experiment [19] revealed that the addition of DRS (without selenization) to up to 14% of the total diet did not affect the hematological parameters of rats, regardless of the potential antinutritional characteristics of DRS. Svetina et al. [49] observed no changes in the hematological parameters of pigs fed with a diet containing up to 10% DRS. Similar findings were published by Trávníček et al. (1995), using sheep as the study animal. Therefore, no adverse effect of DRS addition to the rat diet on the main hematological parameters was observed. Similarly, no changes in hematological parameters were observed in goats when they were fed on a diet supplemented with selenium yeast and sodium selenite, resulting in 0.3 mg Se/kg of the diet [50]. Alashi et al. [51] reported antihypertensive activities of rapeseed protein hydrolysates (alcalase and pepsin hydrolysates) in SHR rats. In our case, significantly (*P* < 0.05) higher total erythrocyte count, lower mean cell volume, and lower total leukocyte count were observed in SHR rats compared to Wistar rats. However, these parameters were not affected by the elevated Se intake in this experiment. Tvrdá et al. [19] also observed no effect of increasing nonselenized DRS intake on ALT activity. In this experiment, no effect of enhanced Se and vitamin E intake was observed, although it has been found that vitamin E and nutrient status can affect hepatic enzyme profiles in the case of other elements such as Zn [52]. Thus, our results confirmed a negligible effect of the rat diet modifications on liver function.

Selenium deficiency, or combined iodine and selenium deficiency, can cause significant alterations in the activities of antioxidant enzymes such as GPx, SOD, and CAT in the thyroid, liver, brain, kidneys, and plasma of rats [53]. Additionally, Parshukova et al. [54] reported the effect of low levels of plasma selenium on GPx activity and thyroid hormone levels in humans living in northern European Russia. Thus, an imbalance in Se and/or I status of animals can result in altered activity of the main antioxidative enzymes, including those closely connected with Se metabolism.

Induction of pulmonary hypertension in Sprague-Dawley rats resulted in increased GPx and decreased CAT activity in animal tissues [55]. Similarly, increasing erythrocyte GPx and CAT activity was observed by Cinar et al. [56] in rats with increasing blood pressure. In contrast, lower GPx activity in serum of rats with arterial hypertension was observed by Ciocoiu et al. [57]. Also Lee et al. [58] reported lower GPx activity in kidney of SHR rats compared to Wistar rats. Vericel et al. [59] suggested that decreased GPx activity could induce an increased cellular generation of radical species and lipid peroxidation, which might be linked to hypertension in SHR rats. Ruseva et al. [60] observed that Se supplementation significantly increased GPx activity of whole blood and in the aortas of both Wistar and SHR rats. Moreover, decreased lipid peroxidation level in the aortic wall was found in Se-supplemented SHR rats compared to the nonsupplemented animals. As a result, Se supplementation improved the redox status of the aortic wall in SHR rats. Our previous investigations [19] showed that GPx levels in plasma decreased with increasing nonselenized DRS in the diet, whereas the specific activities of plasmatic GR, GST, TrxR, and CAT increased (*P* < 0.05). These results indicate that the dietary intake of DRS can affect antioxidative enzyme activity, and the slightly increased Se content in the diet, as in this experiment, cannot balance this effect. However, the additional effect of vitamin E seems to be sufficient to increase the antioxidative response of rats. For instance, an increase in the antioxidant status of boar ejaculate was observed by Horký et al. [61] after the simultaneous addition of selenium, vitamin C, and vitamin E to the diet, in rates of 0.5, 350, and 70 mg per kg of diet, respectively. Thus, the increase in Se intake by SHR rats due to ingestion of selenized DRS, at the levels used in our experiment, needs to be supported by another antioxidative agent such as vitamin E.

Mature seeds of oilseed rape are rich in oil (45–50% v/v); the oil from 00-quality oilseed rape contains high amounts of oleic acid (C18:1, 60% v/v), along with moderate amounts linoleic acid (C18:2, ~30% v/v) and linolenic acid (C18:3, ~10% v/v [62]). Similarly, El-Beltagi and Mohamed [63] investigated the variation in fatty acid composition of oilseed rape and found that oleic acid (C18:1) ranged from 56.3% to 58.7%, linoleic acid (C18:2) from 10.5% to 13.7%, *α*-linolenic acid (C18:3) from 8.8% to 10.3%, and erucic acid (22 : 1) from 0.15% to 0.91%. Xun et al. [64] investigated the long-term effect of human diets containing omega-3 polyunsaturated fatty acids on the occurrence of hypertension, revealing that the individuals with the highest intake of these compounds had a significantly lower incidence of hypertension. Moreover, this effect was more pronounced at higher Se levels, as measured in toenail clippings [64]. Ono et al. [65] proved that antihypertensive drugs are associated with improved fatty acid metabolism in SHR rats. Thus, we can assume that antihypertensive effect of Se could also lead to the better utilization of the DRS-derived fatty acids. Previous results [19] showed lower cholesterol content in the plasma of Wistar rats (*P* < 0.05) with increasing nonselenized DRS in the diet. In contrast, increasing cholesterol and triglyceride levels with increasing blood pressure of rats has been reported by Cinar et al. [56]. A decreased cholesterol level in the blood plasma of lambs has been associated with a dietary supplement of Se, Zn, and vitamin E [66]. The relationships among cholesterol level, Se, and vitamin E supplementation in SHR rats were evaluated by Stone et al. [67], where the plasma cholesterol level of the animals fed on the diet containing 1% cholesterol and deficient in both selenium and vitamin E exceeded almost twice the levels of animals fed on the Se and vitamin E supplemented diet. Besides the effect of polyunsaturated fatty acids on cholesterol levels, Ikeda et al. [68] observed a hypocholesterolemic effect of fiber compounds in the diet. In this context, the fiber content in the DRS should be twice as high as in soybean meal [69] and this factor should be taken into account when assessing the potential effects of DRS addition to animal diets.

To conclude the experiments, the application of Se-supplemented diets did not result in any significant change in the essential element levels of Wistar rat tissues, which was in accordance with our previous results [20], and confirmed that DRS and/or selenized DRS dietary intake did not result in any adverse effects on the nutrient status or balance of the animals. More alterations were observed on the case of SHR rats, where significant differences occurred also between the element levels in Wistar and SHR rats due to differing metabolism of the both strains. However, slight shifts in iodine speciation were recorded in animals fed on the selenized diets. In contrast, slight and ambiguous alterations in the antioxidative response of rats were observed, and vitamin E addition to the diet helped to moderate these effects. The influence of DRS on plasma cholesterol content was not significantly contrary to our preliminary observations [19]. In most of the parameters tested, significant (*P* < 0.05) differences were observed between Wistar and SHR rats, regardless of the experimental diet, indicating that the selenized DRS addition to the rat diet was unable to reduce the differences in nutritional and biochemical status between the rat strains. The potential improvement of the Se status in Se-deficient areas has already been intensively investigated. In agreement with previous studies, selenized DRS application at the levels used in this experiment tended to improve the nutritional status of the rat and can be recommended as an alternative to the Se-rich, complementary feedstuffs for farm animals.

## Figures and Tables

**Table 1:** The nutritional values of the experimental diets.

	Diet A	Diet B	Diet C
Crude protein	19.0 g/kg	19.0 g/kg	19.0 g/kg
Crude fat	33 g/kg	33 g/kg	33 g/kg
Crude fiber	49 g/kg	49 g/kg	49 g/kg
Crude ash	64 g/kg	64 g/kg	64 g/kg
Starch	365 g/kg	365 g/kg	365 g/kg
Sugar	47 g/kg	47 g/kg	47 g/kg
Vitamin A	25.000 IU/kg	25.000 IU/kg	25.000 IU/kg
Vitamin D_3_	1.500 IU/kg	1.500 IU/kg	1.500 IU/kg
Vitamin E	125 mg/kg	125 mg/kg	161 mg/kg
Vitamin K3	20 mg/kg	20 mg/kg	20 mg/kg

Soybean meal	140 g/kg	—	—
DRS	—	140 g/kg	140 g/kg

**Table 2:** The element contents in the experimental diets (mg/kg of dry matter); the averages marked by the same letter did not significantly differ at *P* < 0.05 within individual rows; data are presented as mean ± standard deviation, *n* = 3.

	Diet A	Diet B	Diet C
Se	0.082 ± 0.014^a^	0.184 ± 0.001^b^	0.230 ± 0.001^b^
I	3.45 ± 0.88^a^	2.85 ± 0.88^a^	2.85 ± 1.07^a^
Ca	11146 ± 393^a^	10188 ± 766^a^	10305 ± 79^a^
Cu	11.5 ± 0.2^a^	10.5 ± 0.6^a^	10.3 ± 1.7^a^
Fe	206 ± 25^a^	233 ± 41^a^	210 ± 14^a^
K	6694 ± 774^a^	8270 ± 74^ab^	8925 ± 88^b^
Mg	1876 ± 24.2^a^	2158 ± 256^a^	1905 ± 37.9^a^
Mn	49.2 ± 1.9^a^	60.4 ± 4.3^a^	51.6 ± 0.1^a^
P	6133 ± 280^a^	6303 ± 849^a^	5503 ± 89^a^
S	1809 ± 11^a^	2104 ± 260^a^	1868 ± 38^a^
Zn	60.4 ± 2.43^a^	58.0 ± 5.7^a^	50.2 ± 0.4^a^

**Table 3:** The element contents in whole blood of rats (mg/L); the averages marked by the same letter did not significantly differ at *P* < 0.05 within individual rows for individual rat strains; data are presented as mean ± standard deviation, *n* = 8.

	Wistar rats			SHR rats		
	Diet A	Diet B	Diet C	Diet A	Diet B	Diet C
Se	0.252 ± 0.021^a^	0.263 ± 0.048^a^	0.307 ± 0.029^a^	0.303 ± 0.026^a^	0.338 ± 0.032^ab^	0.363 ± 0.032^b^
Ca	39.5 ± 2.1^a^	43.4 ± 4.6^ab^	50.7 ± 6.1^b^	67.8 ± 31.3^b^	41.8 ± 8.3^ab^	32.5 ± 3.9^a^
Cu	0.891 ± 0.058^a^	0.927 ± 0.815^a^	1.07 ± 0.12^b^	0.505 ± 0.088^b^	0.418 ± 0.069^ab^	0.335 ± 0.043^a^
Fe	460 ± 33^a^	447 ± 59^a^	469 ± 31^a^	392 ± 45^b^	336 ± 28^a^	319 ± 26^a^
K	4383 ± 781^a^	4298 ± 624^a^	4626 ± 312^a^	3042 ± 376^b^	2784 ± 520^ab^	2481 ± 231^a^
Mg	25.5 ± 2.7^a^	24.9 ± 1.6^a^	27.8 ± 2.0^a^	21.7 ± 3.1^b^	17.3 ± 2.3^a^	15.2 ± 1.8^a^
Mn	0.068 ± 0.024^a^	0.072 ± 0.035^a^	0.042 ± 0.011^a^	0.342 ± 0.046^c^	0.240 ± 0.031^b^	0.190 ± 0.020^a^
P	316 ± 30^ab^	299 ± 30^b^	323 ± 34^ab^	315 ± 31^b^	277 ± 20^a^	267 ± 20^a^
S	1299 ± 82^a^	1299 ± 77^a^	1490 ± 100^a^	1296 ± 99^a^	1249 ± 65^a^	1249 ± 521^a^
Zn	3.54 ± 0.23^a^	3.45 ± 0.51^a^	3.85 ± 0.41^a^	5.27 ± 1.43^b^	3.67 ± 0.50^a^	3.08 ± 0.31^a^

**Table 4:** The element contents in rat liver (mg/kg of dry matter); the averages marked by the same letter did not significantly differ at *P* < 0.05 within individual rows for individual rat strains; data are presented as mean ± standard deviation, *n* = 8.

	Wistar rats			SHR rats		
	Diet A	Diet B	Diet C	Diet A	Diet B	Diet C
Se	1.75 ± 0.25^a^	1.98 ± 0.49^a^	1.92 ± 0.22^a^	1.85 ± 0.23^a^	2.46 ± 0.63^a^	2.15 ± 0.33^a^
I	0.128 ± 0.019^a^	0.109 ± 0.019^a^	0.121 ± 0.027^a^	0.095 ± 0.015^a^	0.102 ± 0.025^a^	0.093 ± 0.034^a^
Ca	123 ± 17^a^	118 ± 15^a^	129 ± 24^a^	111 ± 26^a^	106 ± 27^a^	102 ± 27^a^
Cu	12.4 ± 1.0^a^	12.5 ± 1.9^a^	12.9 ± 0.7^a^	9.69 ± 1.52^a^	9.0 ± 2.2^a^	8.7 ± 2.0^a^
Fe	435 ± 65^a^	430 ± 66^a^	457 ± 63^a^	236 ± 38^a^	204 ± 53^a^	205 ± 58^a^
K	10623 ± 860^a^	10728 ± 480^a^	11191 ± 1050^a^	10925 ± 1130^a^	12176 ± 1570^a^	10970 ± 661^a^
Mg	607 ± 63^a^	567 ± 84^a^	625 ± 71^a^	515 ± 94^a^	493 ± 117^a^	462 ± 112^a^
Mn	5.98 ± 0.80^a^	5.68 ± 0.84^a^	6.12 ± 0.48^a^	6.62 ± 0.81^a^	6.41 ± 1.35^a^	6.29 ± 1.08^a^
P	8418 ± 849^a^	8567 ± 581^a^	8965 ± 1035^a^	7839 ± 1207^a^	7670 ± 1548^a^	7207 ± 1505^a^
S	6114 ± 727^a^	6208 ± 407^a^	6566 ± 692^a^	5893 ± 605^a^	6031 ± 899^a^	5788 ± 1008^a^
Zn	68.1 ± 2.9^a^	65.6 ± 1.2^a^	72.7 ± 2.3^b^	58.9 ± 10.7^a^	63.9 ± 17.4^a^	52.9 ± 13^a^

**Table 5:** The element contents in rat kidney (mg/kg of dry matter); the averages marked by the same letter did not significantly differ at *P* < 0.05 within individual rows for individual rat strains; data are presented as mean ± standard deviation, *n* = 8.

	Wistar rats			SHR rats		
	Diet A	Diet B	Diet C	Diet A	Diet B	Diet C
Se	3.74 ± 0.32^a^	3.52 ± 0.25^a^	3.53 ± 0.88^a^	4.52 ± 0.47^ab^	4.32 ± 0.43^a^	4.99 ± 0.52^b^
Ca	324 ± 39^a^	283 ± 42^a^	286 ± 32^a^	162 ± 36^a^	179 ± 27^a^	158 ± 49^a^
Cu	34.8 ± 10.8^a^	33.2 ± 3.2^a^	26.8 ± 4.1^a^	18.6 ± 2.8^a^	19.6 ± 2.9^a^	18.4 ± 3.4^a^
Fe	255 ± 27^a^	284 ± 42^a^	315 ± 116^a^	124 ± 29^a^	145 ± 24^a^	136 ± 33^a^
K	10832 ± 624^a^	10909 ± 405^a^	10809 ± 667^a^	14775 ± 5529^a^	13631 ± 1599^a^	12974 ± 1485^a^
Mg	711 ± 65^a^	722 ± 64^a^	689 ± 45^a^	458 ± 83^a^	534 ± 54^a^	471 ± 92^a^
Mn	3.09 ± 0.23^a^	3.24 ± 0.24^a^	3.13 ± 0.29^a^	3.54 ± 0.43^a^	3.71 ± 0.44^a^	3.03 ± 0.60^a^
P	9372 ± 852^a^	9775 ± 763^a^	9669 ± 663^a^	7063 ± 1011^a^	8020 ± 806^a^	7231 ± 1294^a^
S	7948 ± 589^ab^	8561 ± 746^b^	7300 ± 472^a^	5889 ± 652^a^	6693 ± 437^a^	6104 ± 941^a^
Zn	67.5 ± 7.5^a^	70.2 ± 6.4^a^	70.2 ± 6.1^a^	44.7 ± 7.7^a^	51.7 ± 6.3^a^	46.4 ± 8.9^a^

**Table 6:** The element contents in rat testes (mg/kg of dry matter); the averages marked by the same letter did not significantly differ at *P* < 0.05 within individual rows for individual rat strains; data are presented as mean ± standard deviation, *n* = 8.

	Wistar rats			SHR rats		
	Diet A	Diet B	Diet C	Diet A	Diet B	Diet C
Se	2.07 ± 0.15^a^	2.10 ± 0.3^a^	2.44 ± 0.39^a^	4.56 ± 0.27^a^	4.45 ± 0.18^a^	4.59 ± 0.35^a^
Ca	276 ± 46^a^	297 ± 117^a^	296 ± 34^a^	201 ± 20^a^	206 ± 5^a^	216 ± 12^a^
Cu	11.2 ± 1.4^a^	12.0 ± 2.9^a^	11.8 ± 0.7^a^	8.99 ± 0.71^b^	10.2 ± 0.9^a^	10.4 ± 0.5^a^
Fe	177 ± 27^a^	178 ± 44^a^	172 ± 13^a^	82.6 ± 10.7^a^	87.5 ± 4.3^a^	93.0 ± 11.6^a^
K	21656 ± 1756^a^	22152 ± 3485^a^	22381 ± 1111^a^	24796 ± 1501^a^	24691 ± 1577^a^	25267 ± 1712^a^
Mg	810 ± 109^a^	847 ± 161^a^	866 ± 66^a^	758 ± 62^a^	810 ± 26^ab^	844 ± 37^b^
Mn	1.65 ± 0.22^a^	1.69 ± 0.4^a^	1.66 ± 0.14^a^	1.49 ± 0.16^a^	1.51 ± 0.05^a^	1.60 ± 0.10^a^
P	10855 ± 1315^a^	11303 ± 2196^a^	11468 ± 942^a^	10163 ± 693^a^	10582 ± 346^ab^	11121 ± 494^b^
S	6757 ± 857^a^	7141 ± 1628^a^	7314 ± 611^a^	6116 ± 293^a^	6326 ± 192^ab^	6614 ± 289^b^
Zn	129 ± 17^a^	134 ± 25^a^	136 ± 10^a^	121 ± 10^a^	134 ± 18^a^	133 ± 7^a^

**Table 7:** Average contents of main hematological parameters; the averages marked by the same letter did not significantly differ at *P* < 0.05 within individual rat strains, *n* = 8; data are presented as mean ± standard deviation.

	Er (T/L)	Hct (%)	MCV (fL)	Hb (g/100 mL)	Le (G/L)
Wistar rats					
Diet A	7.60 ± 0.36^a^	49.8 ± 1.9^a^	65.6 ± 2.3^a^	139 ± 6^a^	13.6 ± 3.0^a^
Diet B	7.59 ± 0.39^a^	49.0 ± 2.6^a^	64.6 ± 3.2^a^	132 ± 6^a^	13.3 ± 5.3^a^
Diet C	7.67 ± 0.53^a^	48.8 ± 3.7^a^	63.4 ± 1.5^a^	135 ± 7^a^	12.8 ± 3.5^a^
SHR rats					
Diet A	8.50 ± 0.43^a^	48.6 ± 2.9^a^	57.0 ± 2.0^a^	139 ± 8^a^	9.60 ± 1.64^a^
Diet B	8.99 ± 1.05^a^	50.6 ± 6.1^a^	56.3 ± 1.3^a^	137 ± 11^a^	10.8 ± 2.6^a^
Diet C	8.89 ± 0.56^a^	50.1 ± 2.3^a^	56.3 ± 1.7^a^	135 ± 9^a^	8.46 ± 1.20^a^

Er: total erythrocyte count, Hct: hematocrit, MCV: mean cell volume, Hb: hemoglobin, and Le: total leukocyte count.

**Table 8:** Average contents of specific activities of antioxidative enzymes determined in animal tissues; the averages marked by the same letter did not significantly differ at *P* < 0.05 within individual columns, *n* = 8; data are presented as mean ± standard deviation.

	GPx (U/g)	GR (U/g)	GST (U/mg)	TrxR (U/g)	CAT (U/*µ*g)
	Liver				

Wistar rats					
Diet A	27.6 ± 1.6^b^	17.5 ± 2.3^a^	0.31 ± 0.03^a^	4.14 ± 0.46^a^	0.66 ± 0.07^b^
Diet B	26.4 ± 2.4^ab^	25.5 ± 3.3^b^	0.42 ± 0.04^b^	5.15 ± 0.44^b^	0.58 ± 0.05^a^
Diet C	25.5 ± 2.9^a^	32.2 ± 3.0^c^	0.46 ± 0.03^c^	5.23 ± 0.44^b^	0.63 ± 0.07^b^
SHR rats					
Diet A	26.0 ± 1.7^b^	15.7 ± 1.5^a^	0.14 ± 0.01^a^	12.1 ± 1.4^b^	0.59 ± 0.10^b^
Diet B	24.8 ± 1.5^a^	18.0 ± 1.6^b^	0.17 ± 0.01^b^	11.5 ± 1.3^b^	0.56 ± 0.10^a^
Diet C	24.5 ± 1.5^a^	16.2 ± 1.5^a^	0.19 ± 0.01^c^	9.50 ± 1.17^a^	0.56 ± 0.07^a^

	Kidney				

Wistar rats					
Diet A	219 ± 11^ab^	28.0 ± 1.4^b^	0.11 ± 0.01^a^	22.0 ± 1.4^b^	0.32 ± 0.03^a^
Diet B	195 ± 9^a^	22.2 ± 1.7^a^	0.12 ± 0.01^b^	28.6 ± 1.7^c^	0.34 ± 0.03^a^
Diet C	226 ± 10^b^	30.1 ± 1.7^c^	0.13 ± 0.01^c^	18.1 ± 1.5^a^	0.36 ± 0.04^a^
SHR rats					
Diet A	165 ± 11^a^	27.9 ± 2.4^a^	0.05 ± 0.01^a^	20.7 ± 1.3^b^	0.56 ± 0.05^a^
Diet B	245 ± 13^b^	30.8 ± 2.4^b^	0.07 ± 0.01^b^	20.2 ± 1.5^b^	0.58 ± 0.04^a^
Diet C	240 ± 12^b^	47.9 ± 2.5^c^	0.07 ± 0.01^b^	18.5 ± 1.4^a^	0.56 ± 0.04^a^

GPx: glutathione peroxidase, GR: glutathione reductase, GST: glutathione S-transferase, TrxR: thioredoxin reductase, and CAT: catalase.
